# Tricuspid Annular Plane Systolic Excursion-to-Systolic Pulmonary Artery Pressure Ratio as a Prognostic Factor in Heart Transplant Patients

**DOI:** 10.3390/medicina60071078

**Published:** 2024-06-30

**Authors:** Laurentiu Huma, Horatiu Suciu, Calin Avram, Radu-Adrian Suteu, Alina Danilesco, Dragos-Florin Baba, Diana-Andreea Moldovan, Anca-Ileana Sin

**Affiliations:** 1 Emergency Institute for Cardiovascular Diseases and Transplant, 540136 Targu Mures, Romania; laurentiu.huma@umfst.ro (L.H.); horatiu.suciu@umfst.ro (H.S.); radu.suteu@yahoo.com (R.-A.S.); diana.moldovan@umfst.ro (D.-A.M.); 2 Department of Cell and Molecular Biology, “George Emil Palade” University of Medicine, Pharmacy, Science and Technology of Targu Mures, 540142 Targu Mures, Romania; ileana.sin@umfst.ro; 3 Doctoral School, “George Emil Palade” University of Medicine, Pharmacy, Science and Technology of Targu Mures, 540142 Targu Mures, Romania; 4 Department of Surgery, “George Emil Palade” University of Medicine, Pharmacy, Science and Technology of Targu Mures, 540142 Targu Mures, Romania; 5 Department of Medical Informatics and Biostatistics, “George Emil Palade” University of Medicine, Pharmacy, Science and Technology of Targu Mures, 540142 Targu Mures, Romania; 6 Targu Mures County Hospital, 540072 Targu Mures, Romania; alinka942@yahoo.com; 7 Department of Family Medicine, “George Emil Palade” University of Medicine, Pharmacy, Science and Technology of Targu Mures, 540142 Targu Mures, Romania

**Keywords:** heart transplant, TAPSE/sPAP, mortality, survival, complications

## Abstract

*Background and Objectives*: Heart transplant is currently the final step in treating patients with heart failure. The success of this procedure is strongly connected to potential complications such as postoperative heart failure, infections, graft rejection, graft vasculopathy, and kidney failure. Thus, identifying potential prognostic factors for patients’ outcome is of utmost importance. We investigated the prognostic role of the postoperative ratio between the tricuspid annular plane systolic excursion (TAPSE) and systolic pulmonary artery pressure (sPAP) in patients who underwent heart transplantation in our center. *Materials and Methods*: The study included 46 adult patients from the Emergency Institute for Cardiovascular Diseases and Transplant of Târgu Mureș, who underwent heart transplant between January 2011 and April 2023. By the use of receiver operating characteristic (ROC) analysis, we determined an optimal cut-off value for TAPSE/sPAP with regard to survival at 6 months. Differences in central tendencies of baseline characteristics in those who had a value lower than the cut-off value of TAPSE/sPAP and those who presented a value above it were investigated using the corresponding parametric or nonparametric tests. *Results*: A value for TAPSE/sPAP above 0.47 mm/mmHg was associated with 6-month survival (OR: 59.5, CI: 5.7–616.0). No significant differences in central tendencies for baseline characteristics were found between the patients who had a TAPSE/sPAP ratio below the cut-off and those who had a ratio above it. *Conclusions*: The TAPSE/sPAP ratio might prove to be valuable in the early identification of at-risk heart transplant patients. Further prospective studies with larger cohorts are required for validation.

## 1. Introduction

Since the first time it was performed in 1967 by Christiaan Barnard [1], heart transplant has been the final step in the treatment of end-stage heart failure, when all other therapeutic options have reached their limit. The procedure requires a series of compatibility tests between the donor and recipient, to ensure the safety of the latter and to help in the prevention of graft rejection. In order to evaluate these conditions, blood samples are collected to identify blood type and baseline laboratory results, the level of inflammatory biomarkers, as well as to screen for infection sources. Unfortunately, there is a large discrepancy between the number of patients who would benefit from a heart transplant and the number of available organs [2]. Appropriate selection of recipients has become one of the major research directions of the past few decades. Different scores have been developed to ensure correct selection, and combining these scores has proven an even better indicator for adequate choice of recipients [3].

After performing the heart transplant, maximizing the chances of the patient’s survival and avoiding postoperative complications in the following stages has to become the target of the medical management. Overall survival, infections, graft dysfunction, acute cellular and antibody-mediated rejection, cardiac allograft vasculopathy, and renal dysfunction are complications that are thoroughly researched, both as singular entities and as part of predictive algorithms of outcome [4].

Various invasive and non-invasive parameters have been tested for prognostic value in the survival of recipients of a transplanted heart or in postoperative complications [5,6,7], but very few have proven their utility in day-to-day clinical use. A few reasons for this are the limited availability of testing kits for newly discovered molecules that may predict outcome [8], difficulty in applying the formulas used without an appropriate computational system, and lack of equipment for performing advanced imaging techniques such as global longitudinal strain (GLS) of the left and right ventricles in heart transplant recipients [9].

Considering the fact that clinicians usually have access to two-dimensional (2D) echocardiography, it is fundamental to search for diagnostic and prognostic tools that could have their foundation in this investigation. The potential of echocardiography is not limited to its solitary use, but information provided through it might also be connected to different signs or prognostic tools provided by existing technology (e.g., electrocardiography, right heart catheterization, cardiac biopsies, serum biomarkers, etc.).

Novel but broadly available markers have to be investigated in heart transplant patients in order to ensure high-quality and uniform care for this special category of individuals. While prognostic markers of left ventricular (LV) function tend to detach from the widely accessible measurements, focusing on global longitudinal strain and other advanced measurements [9], markers of the right ventricular (RV) function have not yet been validated in certain clinical scenarios, as is the case for heart transplant. Lately, the emphasis was put on the ratio between the tricuspid annular plane systolic excursion (TAPSE) and systolic pulmonary artery pressure (sPAP) as an interesting predictor of outcomes in various diseases, such as heart failure, pulmonary hypertension, systemic sclerosis, and amyloidosis [10,11,12,13,14]. 

The TAPSE/sPAP ratio is an important marker of ventriculo-arterial coupling, as the RV is exposed to hemodynamic burdens, affecting its compliance, with an impact on its overall performance. Thus, RV-PA coupling refers to the relationship between RV contractility and RV afterload. To maintain the normal coupling, the pulmonary vascular resistance and RV function must be appropriately matched. Uncoupling of the RV-PA occurs either when the RV function cannot increase to match the afterload, or in the case of a decrease in RV function with constant or increased pulmonary vascular resistance. In the early stages post-heart transplant, this relationship is mainly influenced by the RV function, with great influence on the patients’ prognosis [15,16]. There is an increasing number of studies that show the pivotal role of RV-PA coupling in heart failure, highlighted through the TAPSE/sPAP ratio, by correlating different cut-off values of the marker with mortality, hospitalization rates, exercise capacity, and postoperative complications [17,18].

The aim of this study was to investigate a potential relationship between the postoperative TAPSE/sPAP ratio in patients who underwent heart transplant and survival at 6 months. We determined the best-suited cut-off value by using receiver operating characteristic (ROC) analysis and the maximum Youden’s index, while investigating a possible association between the threshold and 6-month survival. Thus, a significant result of our study may bring improvements to the management algorithm of heart transplant patients while relying on a widely accessible, cost-efficient, easy to determine echocardiographic marker.

## 2. Materials and Methods

**Figure 1:**
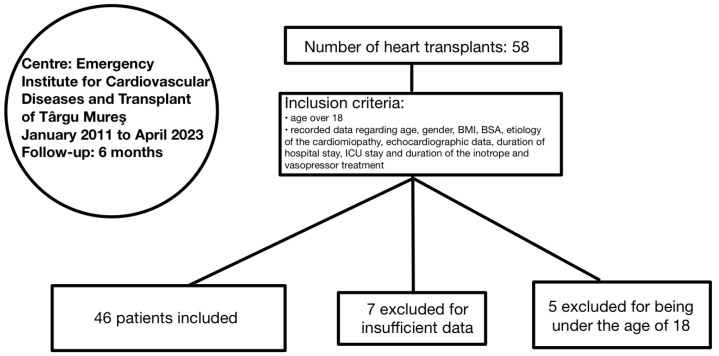
Inclusion criteria of our study. BMI: body mass index; BSA: body surface area; ICU: intensive care unit.

The study involved adult patients admitted to the Emergency Institute for Cardiovascular Diseases and Transplant of Târgu Mureș who underwent heart transplant during the period from January 2011 to April 2023. The initial cohort was made up of 58 patients, out of which 7 were excluded for incomplete data records and 5 of them for being under the age of 18 (Figure 1). Informed consent was obtained from the involved participants. The study protocol was approved by the ethics committee of the Emergency Institute of Cardiovascular Diseases and Transplant of Târgu Mureș and the research was conducted in accordance with the Helsinki Declaration.

Baseline data were collected for all patients, including age, gender, weight, body mass index (BMI), body surface area (BSA), ischemic or non-ischemic etiology of the underlying cardiomyopathy, duration of the hospital stay, intensive care unit (ICU) stay, and duration of intravenous (i.v.) inotrope/vasopressor treatment. Echocardiographic data were collected from echocardiographic protocols, as well as the sheets provided during the patient’s stay in the ICU. For data regarding postoperative echocardiographic measurements, the highest value recorded during the postoperative hospital stay was selected. The 6-month survival was assessed by data collected from the datasheets available in the intranet system of the Emergency Institute for Cardiovascular Diseases and Transplant of Târgu Mureș.

The statistical analysis was performed using MedCalc version 22 (MedCalc Software Ltd., Ostend, Belgium). For quantitative data we determined maximum, minimum, median, mean values, and standard deviations (SD). The test chosen for evaluating the normal distribution of values was the Shapiro–Wilk test. In order to analyze the relationship between postoperative TAPSE/sPAP ratio and 6-month survival in our cohort, we determined a cut-off value for TAPSE/SPAP by the use of ROC curves and the maximum Youden’s index. Afterwards, an association between patients who had a value above our cut-off value and survival was investigated using Fischer’s exact test. The significance threshold was set to 0.05.

**Figure 2:**
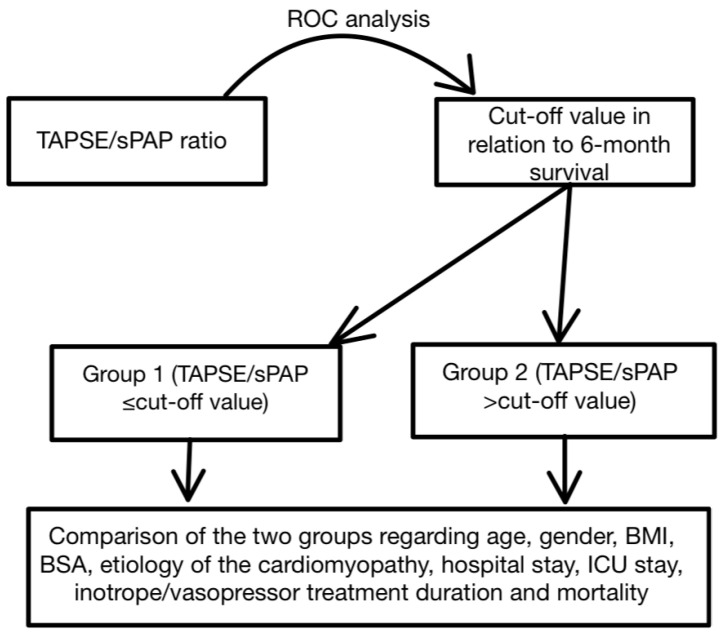
Stage two of the study—comparing central tendencies. ROC: receiver operating characteristic; TAPSE: tricuspid annular plane systolic excursion; sPAP: systolic pulmonary artery pressure. BMI: body mass index; BSA: body surface area; ICU: intensive care unit.

In the second stage of our investigation, we divided the cohort into two groups with regard to their relation to the previously determined cut-off value and compared central tendencies between the groups for age, BMI, BSA, total length of hospital stay, duration of ICU stay, and duration of inotrope/vasopressor treatment by the use of parametric or nonparametric tests (Figure 2).

## 3. Results

When looking at the baseline characteristics of our cohort we included 46 patients, out of which 6 were females (13.04%) and 40 were males (86.96%). The mean age for our group was 45.19 years (SD = 10.50), with an interval ranging from 18 to 61 years. Regarding BMI, the mean value was 24.78 kg/sqm (SD = 4.08), while the mean value for BSA was 1.92 sqm (SD = 0.22). Prior to the heart transplant, 45 of the 46 patients had a severely reduced left ventricle ejection fraction (LVEF) of under 30%, while one of the patients had end-stage heart failure secondary to a predominantly right-chambers cardiomyopathy. A total of 34 (73.91%) patients had a non-ischemic etiology of the cardiomyopathy, while 12 (26.09%) were characterized by an ischemic origin of the disease.

Out of the echocardiographic findings, after the heart transplant, a mean value of 17.06 mm (SD = 3.17) was identified for TAPSE, with a mean sPAP value of 32.67 mmHg (SD = 7.90). Regarding the TAPSE/sPAP ratio, the mean value was 0.56 mm/mmHg (SD = 0.19).

The mean duration of hospitalization was 56.89 days, with a mean ICU stay of 49.23 days. The mean time interval when patients required inotrope or vasopressor i.v. treatment was 8.39 days (Table 1). The 6-month mortality of our cohort was 17.39% (8:46 patients).

**Figure 3:**
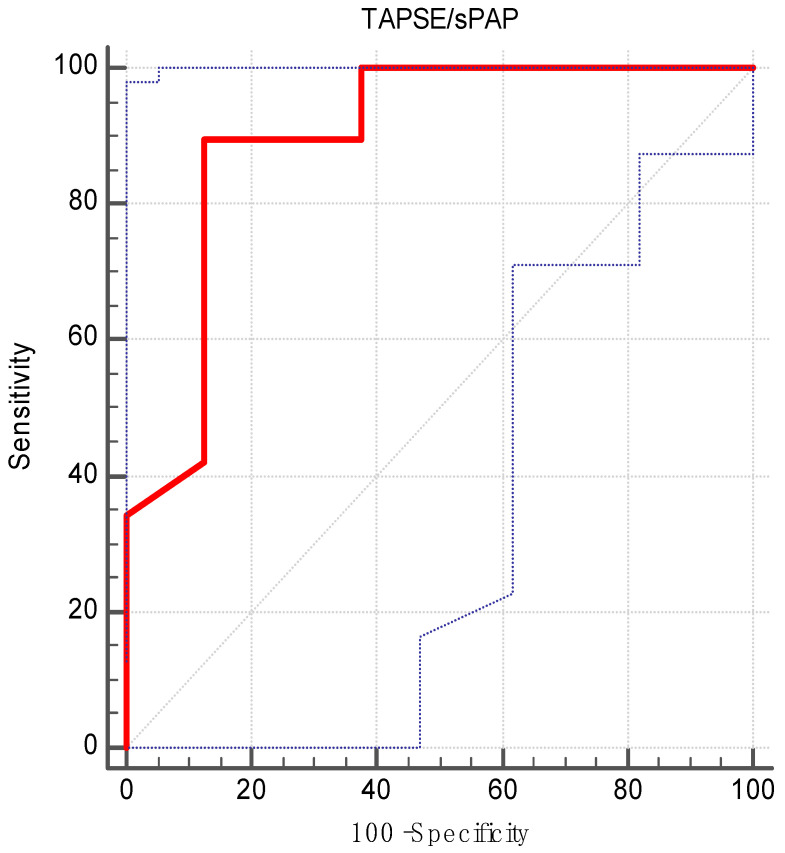
Predictive value of TAPSE/sPAP ratio for 6-month survival. TAPSE: tricuspid annular plane systolic excursion; sPAP: systolic pulmonary artery pressure.

Using ROC analysis and the maximum Youden index (Figure 3), an optimal cut-off value for postoperative TAPSE/sPAP ratio of 0.47 mm/mmHg was determined in relation to 6-month survival. This corresponded to an area under the curve (AUC) of 0.89 (CI: 0.77–0.96), with a *p* value of <0.001 (Table 2). This cut-off value of TAPSE/sPAP > 0.47 mm/mmHg was associated with a sensitivity of 89.47% and a specificity of 87.50% (Table 3).

After determining the optimal cut-off value, its statistical significance for 6-month survival was investigated. The cohort was divided into two groups in regard to the cut-off value for TAPSE/sPAP. Out of the total number of patients, 23.91% (11:46 patients) had a TAPSE/sPAP ratio under the cut-off value, while 76.09% (35:46 patients) had a value of over 0.47 mm/mmHg. The first group was characterized by a 6-month mortality of 63.63% (7:11 patients), while the second group had a mortality of 2.85% (1:35) after the heart transplant. By the use of contingency tables and Fisher’s exact test, a significant association was demonstrated between survival at 6 months after heart transplantation and a value of TAPSE/sPAP > 0.47 mm/mmHg, with an odds ratio (OR) of 59.5 (CI: 5.7–616.0, *p* < 0.001) (Table 4). 

When looking at the patients’ gender, group 1 (TAPSE/sPAP ≤ 0.47 mm/mmHg) was made up of 90.90% (10:11) males and 9.10% (1:11) females, while group 2 (TAPSE/sPAP > 0.47 mm/mmHg) had a proportion of 85.71% (30:35) males and 14.29% (5:35) females. The non-ischemic etiology of the cardiomyopathy was characteristic for 81.81% (9:11) of the patients in group 1 with the ischemic etiology making up 18.19% (2:11). Regarding group 2, 71.42% (25:35) of patients had a non-ischemic etiology, while 28.58% (10:35) had an ischemic etiology of the cardiomyopathy. The descriptive statistics of the parameters in the two new groups showed a mean/median value of 1.93 (SD = 0.23)/1.96 sqm for BSA in group 1 and 1.92 (SD = 0.23)/1.96 sqm for group 2. In terms of BMI, a mean/median value of 24.39 (SD = 4.46)/24.6 kg/sqm was recorded for group 1, and 24.90 (SD = 4.02)/24.6 kg/sqm for group 2. The mean age for group 1 was 40.45 (SD = 13.31) years with a median of 37 years, while for group 2 a mean of 46.68 (SD = 9.18) years with a median of 47 years was recorded. When looking at the duration of the hospital stay, a mean of 36.36 (SD = 21.75) days and a median of 32 days was recorded for group 1, while a mean of 63.34 (SD = 69.05) days and a median of 39 days characterized group 2. The ICU stay for group 1 had a mean/median duration of 32.43 (SD = 21.11)/ 32 days and 54.85 (SD = 69.05)/35 days for group 2. Regarding the duration of the i.v. inotrope and vasopressor treatment, group 1 had a mean/median time of 14.36 (SD = 22.59)/5 days while group 2 had a mean/median of 6.51 (SD = 7.24)/4 days (Table 4).

In order to investigate significant differences in the baseline characteristics between the two new groups regarding central tendencies (means or medians), the corresponding statistical tests were applied. Beforehand, the parameters were investigated in regard to their relation to a normal distribution by using the Shapiro–Wilk test (Table 4).

The second step was to apply the suitable statistical test to each one of the parameters. Considering the results of the normality test, the means for age in the two groups were compared by the two-sample *t*-test and a *p* = 0.08 value was obtained. Medians for BMI, BSA, the hospital stay, ICU stay, and time under i.v. inotropes/vasopressors were compared between the two groups using the Mann–Whitney test for nonparametric data. These returned no statistically significant results (*p* = 0.65 for BMI; *p* = 0.84 for BSA; *p* = 0.28 for hospital stay; *p* = 0.27 for length of stay in the ICU; and *p* = 0.06 for time under i.v. inotropes/vasopressors). By applying Fisher’s exact test, we found no significant association between gender and values above the cut-off value for TAPSE/sPAP, nor between the etiology of the cardiomyopathy and having a value of TAPSE/sPAP > 0.47 mm/mmHg.

## 4. Discussion

Heart transplant, as a treatment option for end-stage heart failure, is an imperative area of interest for physicians all over the world. The main reason for this is the curative potential of the procedure in case of excellent donor–recipient selection, optimal periprocedural and long-term treatment, as well as a good understanding of the pathophysiological mechanisms. Poor management is associated with life-threatening complications such as acute postoperative heart failure, infections, acute graft rejection, graft vasculopathy, acute kidney injury, and arrythmias [19]. Thus, identifying markers that could potentially highlight the initial stages of complications and showcase an alarm signal for a high probability of complications is of utmost relevance.

By validating the echocardiographic description of TAPSE as a marker of RV dysfunction, scientists have opened the path for the development of new parameters that showcase right heart function impairment. Lately, clinicians have focused on parameters that highlight more complex processes, such as RV strain through speckle tracking, or RV to PA coupling, through the ratio between TAPSE and the sPAP, estimated by the means of continuous-wave Doppler. As a physiological mechanism, this phenomenon depicts the relationship between RV contractility and RV afterload, with clinical equivalents in RV function and pulmonary vascular resistance, respectively. When RV function decreases and pulmonary vascular resistance is normal or high, there is a mismatch in RV-PA coupling, a phenomenon validated for its prognostic role in heart failure and other diseases [15,16] and influencing the rate of hospital readmission and exercise capacity [17,18]. This newly developed parameter has become an important point of interest through its cost-effectiveness and broad implications as a prognostic tool.

Several studies have investigated the TAPSE/sPAP ratio as a prognostic factor for different conditions in a variety of medical fields, highlighting its use and versatility. Analyzing the EUSTAR cohort, one study established that the TAPSE/sPAP ratio, at a cut-off value of 0.32 mm/mmHg, was a reliable predictor of mortality, while a value of under 0.55 mm/mmHg was associated with the diagnostic of pulmonary hypertension in patients suffering from systemic sclerosis [20]. Moreover, the TAPSE/sPAP ratio has been shown to provide value in advanced selection of patients with a diagnosis of systemic sclerosis for right heart catheterization who initially underwent the DETECT algorithm for pulmonary hypertension [21]. Furthermore, reduced RV-PA coupling, defined by a TAPSE/sPAP ratio of under 0.55 mm/mmHg has been associated with a higher incidence of in-hospital major adverse cardiac events (MACEs) in patients admitted for acute coronary syndromes [22]. TAPSE/sPAP has also been shown to provide prognostic value in patients suffering from valvular disease, more specifically, in tricuspid regurgitation, with many of these studies supporting its use in clinical practice [23,24,25].

In our study, we explored the role of the postoperative TAPSE/sPAP ratio as a prognostic factor for survival in heart transplant patients. Firstly, we performed an ROC analysis considering all the individual values of TAPSE/sPAP and survival. By using the maximum Youden index, we obtained an optimal cut-off value of >0.47 mm/mmHg. This threshold corresponded to a sensitivity of 89.47% and a specificity of 87.50%. Afterwards, we split our cohort into two groups considering their relation to the newly found cut-off value. By applying the statistical analysis, we determined that having a TAPSE/sPAP ratio above 0.47 mm/mmHg was associated with survival at six months after the surgery. 

In order to explore any significant differences in central tendencies regarding baseline characteristics in patients on both sides of the cut-off value for TAPSE/sPAP, we performed the corresponding parametric or nonparametric tests for each one of the variables. By using the Mann–Whitney test for nonparametric data, and Student’s *t*-test for parametric data, no significant differences between central tendencies of the two groups were found regarding age, BMI, BSA, length of hospital stay, length of stay in the ICU, and duration of i.v. inotropes/vasopressors. We investigated a potential association between the etiology of the cardiomyopathy and the ratio of TAPSE/sPAP, as well as for patient’s gender and TAPSE/sPAP by the use of Fisher’s exact test, with no significant result.

The cause of death during the first 6 months after the transplant in our cohort was represented by advanced heart failure developed either during the first hospital admission or during a secondary admission for this particular reason. The patients’ status altered up to the point of multiple system organ failure (MSOF) and death, thus highlighting the pivotal role of markers for early detection of heart failure, among which markers for RV-PA coupling and RV function are included [15,16]. 

Similarly, researchers have investigated different echocardiographic parameters in heart transplant patients, such as markers of diastolic dysfunction (e.g., the relation between E and A flow velocities in relation to tissue Doppler parameters, such as E’), with an emphasis on their prognostic value [26,27,28]. Some studies used advanced techniques, such as longitudinal strain by speckle tracking of the LV, to assess and compare diastolic dysfunction markers to traditional echocardiographic parameters, with notable results [29]. This investigation carries the burden of scarcity, through the need of expensive software to operate such measurements. Even though it is becoming an increasingly common practice in developed countries, more time is needed for global longitudinal strain to be part of the routine echocardiographic examination worldwide. 

Through the development and highlighting of different non-invasive and invasive potential predictive markers for mortality and complications, such as echocardiographic rejection scores, cardiac magnetic resonance markers, 3D optical coherence tomography, or inflammatory markers levels [30,31,32,33,34,35,36,37,38,39,40], composite prognostic scores should be proposed and investigated for their potentially incremental utility and prevention benefits. By using markers that have proven their utility as prognostic factors in heart transplant that are part of different diagnostic tools (e.g., echocardiography, electrocardiography, right heart catheterization, serum biomarkers, cardiac biopsies, etc.), new prognostic algorithms should be developed and validated through future studies. 

To summarize, it is of utmost importance to explore both novel or refined and reinterpreted diagnostic and prognostic tools. The aim should be to extend the net benefit of the marker while also considering its availability, in order to provide high-quality care to as many patients as possible. Prognostic markers derived from the 2D echocardiographic examination may provide incremental value based on the investigation’s high availability and reproducibility. Moreover, 2D echocardiography is part of routine practice in most cardiology departments, providing a field of study that could include large numbers of patients in case of an extensive study, providing a building ground for future research. The TAPSE/sPAP ratio as a marker of RV to PA coupling has started to rise in popularity in many fields, while being an accessible option for physicians to use in their clinical practice, even though universally applicable cut-off values are yet to be determined.

Lastly, cardiac transplantation is a challenge not only for the physician and the patient, but also for the close relatives of the individual. As a recent review has proven, up to the point of the transplant procedure, spouses of recipients undergo a period characterized by uncertainty, thoughts about death, changes to lifestyle and priorities, loss of sense of self, decrease in quality of life, and learning to cope [41]. Another study [42] has pointed out the relationship between relatives in this all-consuming time frame when the recipient awaits a suitable heart. These concepts increase the importance of a detailed psychological and psychiatric examination of organ recipients, with the need of potential medical intervention in particular cases to improve mental status and outcomes. 

Taking into consideration the conclusions of these studies, identifying new prognostic factors that might change the management algorithm of this category of patients could also have a large impact not only on the life of patients, but on their close relatives’ lives as well. Hospitals should provide end-stage heart failure patients who find themselves on the waiting list for transplant with access to a psychologist specialized in end-stage disease to better understand the emotional implications of their condition and find solutions for a better quality of life.

Limitations of this study should be considered. Differential losses to follow-up, information bias, and absence of data on potential confounding factors may be issued to the retrospective design of the research [43]. Regarding the donors’ organs, lack of legislation regarding the default status of citizens as organ donors and need of informed consent from the donor’s family members in the short time after the death of a close relative are factors that contribute to a low donor pool in Romania. Furthermore, lack of mass-media information campaigns on the potential life-saving implications of organ donation has also contributed to the scarcity of organs to be transplanted [44,45]. The validity of the results may be influenced by the small cohort in our study through generating false positive data, which can be grounds for overestimating associations between events by misinterpretation of CI and statistical significance [43]. In order to further evaluate and validate the usefulness and prognostic value of the TAPSE/sPAP ratio in heart transplant, larger cohorts and prospective studies are needed.

## 5. Conclusions

In our study, the determined cut-off value of over 0.47 mm/mmHg for postoperative TAPSE/sPAP was associated with survival at 6 months in heart transplant patients in our center. When comparing the group of patients who had a TAPSE/sPAP ratio of under 0.47 mm/mmHg and those with a value above that threshold, we found no statistically significant differences in terms of the age, gender, BMI, BSA, etiology of cardiomyopathy, total hospital stay, length of stay in the ICU, and the length of i.v. inotrope/vasopressor treatment.

## Figures and Tables

**Table 1:** Baseline characteristics.

Characteristics	Mean	SD
Age (years)	45.19	10.50
BMI (kg/sqm)	24.78	4.08
BSA (sqm)	1.92	0.22
TAPSE (mm)	17.06	3.17
sPAP (mmHg)	32.67	7.90
TAPSE/sPAP (mm/mmHg)	0.56	0.19
Hospital stay (days)	56.89	65.17
ICU stay (days)	49.23	61.57
i.v. inotropes/vasopressors (days)	8.39	12.82

BMI: body mass index; BSA: body surface area; TAPSE: tricuspid annular plane systolic excursion; sPAP: systolic pulmonary artery pressure; ICU: intensive care unit.

**Table 2:** Area under the curve (AUC) of TAPSE/sPAP.

AUC	0.89
Standard error	0.07
95% CI	0.77–0.96
z statistic	5.11
*p* value	<0.001

**Table 3:** Maximum Youden’s index.

Maximum Youden‘s index	0.76
Associated criterion	>0.47
Sensitivity	89.47
Specificity	87.50

**Table 4:** Baseline characteristics of the two groups (group 1: TAPSE/sPAP ≤ 0.47, group 2: TAPSE/sPAP > 0.47).

Parameter	Group 1 (n = 11)	Group 2 (n = 35)	*p* Value
Age, mean (SD)/median (years)	40.45 (13.31)/37	46.68 (9.18)/47	0.08 *
Gender (male), n (%)	10 (90.90)	30 (85.71)	1.00 †
BMI, mean (SD)/median (kg/sqm)	24.39 (4.46)/24.6	24.90 (4.02)/24.6	0.65 **
BSA, mean (SD)/median (sqm)	1.93 (0.23)/1.96	1.92 (0.23)/1.96	0.84 **
Non-ischemic cardiomyopathy, n (%)	9 (81.81)	25 (71.42)	0.70 †
Hospital stay, mean (SD)/median (days)	36.36 (21.75)/37	63.34 (72.82)/39	0.28 **
ICU stay, mean (SD)/median (days)	32.54 (21.11)/32	54.85 (69.05)/35	0.27 **
i.v. inotropes/vasopressors, mean (SD)/median (days)	14.36 (22.59)/5	6.51 (7.24)/4	0.06 **
Mortality, n (%)	7 (63.63)	1 (2.85)	<0.001 †

* Two-sample *t*-test assuming equal variances; ** Mann–Whitney test; † Fisher’s exact test.

## Data Availability

All the data generated or analyzed during this study are included in this published article.
